# Supplementary material for: Dissecting the Biological Functions of Various Isoforms of Ferredoxin Reductase for Cell Survival and DNA Damage Response
Source: Cells. 2025 Dec 29;15(1):62. doi: 10.3390/cells15010062 (PMC12785329; doi:10.3390/cells15010062)

Supplemental Figure S1

```
Iso 1 MASRCWRWWGWSAWPRTRLPPAGSTPSFCH------------------------------     30
Iso 2 MASRCWRWWGWSAWPRTRLPPAGSTPSFCH------------------------------     30
Iso 3 MASRCWRWWGWSAWPRTRLPPAGSTPSFCH------------------------------     30
Iso 4 MASRCWRWWGWSAWPRTRLPPAGSTPTFGGSDEVRDPANAKALRNKRRRMQVRVKLGKFQ     60
Iso 5 MASRCWRWWGWSAWPRTRLPPAGSTPSFCH------------------------------     30
Iso 6 MASRCWRWWGWSAWPRTRLPPAGSTPSFCH------------------------------     30
Iso 7 ------------------------------------------------------------     0
      ............................

Iso 1 -HFSTQEKTPQICVVGSGPAGFYTAQHLLK------------------------------     59
Iso 2 -HFSTQEKTPQICVVGSGPAGFYTAQHLLK------------------------------     59
Iso 3 -HFSTQEKTPQICVVGSGPAGFYTAQHLLKRVEALCSQPRVLNSPALSGEGEDLGASQPL     89
Iso 4 LLLDIQEKTPQICVVGSGPAGFYTAQHLLK------------------------------     90
Iso 5 -HFSTQEKTPQICVVGSGPAGFYTAQHLLK------------------------------     59
Iso 6 -HFSTQEKTPQICVVGSGPAGFYTAQHLLKQ-----------------------------     60
Iso 7 ------------------------------------------------------------     0
           .........................

Iso 1 -------------HPQAHVDIYEKQPVPFGLVRFGVAPDHPEVKNVINTFTQTAHSGRCA     106
Iso 2 -------------HPQAHVDIYEKQPVPFGLVRFGVAPDHPEVKNVINTFTQTAHSGRCA     106
Iso 3 SLDPTSCHPVPQQHPQAHVDIYEKQPVPFGLVRFGVAPDHPEVKNVINTFTQTAHSGRCA     149
Iso 4 -------------HPQAHVDIYEKQPVPFGLVRFGVAPDHPEVKNVINTFTQTAHSGRCA     137
Iso 5 -------------HPQAHVDIYEKQPVPFGLVRFGVAPDHPEVK--------TAHSGRCA     98
Iso 6 -------------HPQAHVDIYEKQPVPFGLVRFGVAPDHPEVK----------------     91
Iso 7 ------MEDKDREHPQAHVDIYEKQPVPFGLVRFGVAPDHPEVKNVINTFTQTAHSGRCA     54
                   *******************************................

Iso 1 FWGNVEVGRDVTVPELREAYHAVVLSYGAEDHRALEIPGEELPGVCSARAFVGWYNGLPE     166
Iso 2 FWGNVEVGRDVTVPELREAYHAVVLSYGAEDHRALEIPGEELPGVCSARAFVGWYNGLPE     166
Iso 3 FWGNVEVGRDVTVPELREAYHAVVLSYGAEDHRALEIPGEELPGVCSARAFVGWYNGLPE     209
Iso 4 FWGNVEVGRDVTVPELREAYHAVVLSYGAEDHRALEIPGEELPGVCSARAFVGWYNGLPE     197
Iso 5 FWGNVEVGRDVTVPELREAYHAVVLSYGAEDHRALEIPGEELPGVCSARAFVGWYNGLPE     158
Iso 6 -------------------------SYGAEDHRALEIPGEELPGVCSARAFVGWYNGLPE     126
Iso 7 FWGNVEVGRDVTVPELREAYHAVVLSYGAEDHRALEIPGEELPGVCSARAFVGWYNGLPE     114
      .........................***********************************

Iso 1 NQELEPDLSCDTAVILGQGNVALDVARILLTPPEHLE------RTDITKAALGVLRQSRV     220
Iso 2 NQELEPDLSCDTAVILGQGNVALDVARILLTPPEHLEALLLCQRTDITKAALGVLRQSRV     226
Iso 3 NQELEPDLSCDTAVILGQGNVALDVARILLTPPEHLE------RTDITKAALGVLRQSRV     263
Iso 4 NQELEPDLSCDTAVILGQGNVALDVARILLTPPEHLE------RTDITKAALGVLRQSRV     251
Iso 5 NQELEPDLSCDTAVILGQGNVALDVARILLTPPEHLE------RTDITKAALGVLRQSRV     212
Iso 6 NQELEPDLSCDTAVILGQGNVALDVARILLTPPEHLE------RTDITKAALGVLRQSRV     180
Iso 7 NQELEPDLSCDTAVILGQGNVALDVARILLTPPEHLE------RTDITKAALGVLRQSRV     168
      *************************************      *****************
```

Supplemental Figure S1
(Continued)

```
Iso 1 KTVWLVGRRGPLQVAFTIKELREMIQLPGARPILDPVDFLGLQDKIKEVPRPRKRLTELL      280
Iso 2 KTVWLVGRRGPLQVAFTIKELREMIQLPGARPILDPVDFLGLQDKIKEVPRPRKRLTELL      286
Iso 3 KTVWLVGRRGPLQVAFTIKELREMIQLPGARPILDPVDFLGLQDKIKEVPRPRKRLTELL      323
Iso 4 KTVWLVGRRGPLQVAFTIKELREMIQLPGARPILDPVDFLGLQDKIKEVPRPRKRLTELL      311
Iso 5 KTVWLVGRRGPLQVAFTIKELREMIQLPGARPILDPVDFLGLQDKIKEVPRPRKRLTELL      272
Iso 6 KTVWLVGRRGPLQVAFTIKELREMIQLPGARPILDPVDFLGLQDKIKEVPRPRKRLTELL      240
Iso 7 KTVWLVGRRGPLQVAFTIKELREMIQLPGARPILDPVDFLGLQDKIKEVPRPRKRLTELL      228
      ************************************************************

Iso 1 LRTATEKPGPAEAARQASASRAWGLRFFRSPQQVLPSPDGRRAAGVRLAVTRLEGVDEAT      340
Iso 2 LRTATEKPGPAEAARQASASRAWGLRFFRSPQQVLPSPDGRRAAGVRLAVTRLEGVDEAT      346
Iso 3 LRTATEKPGPAEAARQASASRAWGLRFFRSPQQVLPSPDGRRAAGVRLAVTRLEGVDEAT      383
Iso 4 LRTATEKPGPAEAARQASASRAWGLRFFRSPQQVLPSPDGRRAAGVRLAVTRLEGVDEAT      371
Iso 5 LRTATEKPGPAEAARQASASRAWGLRFFRSPQQVLPSPDGRRAAGVRLAVTRLEGVDEAT      332
Iso 6 LRTATEKPGPAEAARQASASRAWGLRFFRSPQQVLPSPDGRRAAGVRLAVTRLEGVDEAT      300
Iso 7 LRTATEKPGPAEAARQASASRAWGLRFFRSPQQVLPSPDGRRAAGVRLAVTRLEGVDEAT      288
      ************************************************************

Iso 1 RAVPTGDMEDLPCGLVLSSIGYKSRPVDPSVPFDSKLGVIPNVEGRVMDVPGLYCSGWVK      400
Iso 2 RAVPTGDMEDLPCGLVLSSIGYKSRPVDPSVPFDSKLGVIPNVEGRVMDVPGLYCSGWVK      406
Iso 3 RAVPTGDMEDLPCGLVLSSIGYKSRPVDPSVPFDSKLGVIPNVEGRVMDVPGLYCSGWVK      443
Iso 4 RAVPTGDMEDLPCGLVLSSIGYKSRPVDPSVPFDSKLGVIPNVEGRVMDVPGLYCSGWVK      431
Iso 5 RAVPTGDMEDLPCGLVLSSIGYKSRPVDPSVPFDSKLGVIPNVEGRVMDVPGLYCSGWVK      392
Iso 6 RAVPTGDMEDLPCGLVLSSIGYKSRPVDPSVPFDSKLGVIPNVEGRVMDVPGLYCSGWVK      360
Iso 7 RAVPTGDMEDLPCGLVLSSIGYKSRPVDPSVPFDSKLGVIPNVEGRVMDVPGLYCSGWVK      348
      ************************************************************

Iso 1 RGPTGVIATTMTDSFLTGQMLLQDLKAGLLPSGPRPGYAAIQALLSSRGVRPVSFSDWEK      460
Iso 2 RGPTGVIATTMTDSFLTGQMLLQDLKAGLLPSGPRPGYAAIQALLSSRGVRPVSFSDWEK      466
Iso 3 RGPTGVIATTMTDSFLTGQMLLQDLKAGLLPSGPRPGYAAIQALLSSRGVRPVSFSDWEK      503
Iso 4 RGPTGVIATTMTDSFLTGQMLLQDLKAGLLPSGPRPGYAAIQALLSSRGVRPVSFSDWEK      491
Iso 5 RGPTGVIATTMTDSFLTGQMLLQDLKAGLLPSGPRPGYAAIQALLSSRGVRPVSFSDWEK      452
Iso 6 RGPTGVIATTMTDSFLTGQMLLQDLKAGLLPSGPRPGYAAIQALLSSRGVRPVSFSDWEK      420
Iso 7 RGPTGVIATTMTDSFLTGQMLLQDLKAGLLPSGPRPGYAAIQALLSSRGVRPVSFSDWEK      408
      ************************************************************

Iso 1 LDAEEVARGQGTGKPREKLVDPQEMLRLLGH     491
Iso 2 LDAEEVARGQGTGKPREKLVDPQEMLRLLGH     497
Iso 3 LDAEEVARGQGTGKPREKLVDPQEMLRLLGH     534
Iso 4 LDAEEVARGQGTGKPREKLVDPQEMLRLLGH     522
Iso 5 LDAEEVARGQGTGKPREKLVDPQEMLRLLGH     483
Iso 6 LDAEEVARGQGTGKPREKLVDPQEMLRLLGH     451
Iso 7 LDAEEVARGQGTGKPREKLVDPQEMLRLLGH     439
      *******************************
```

Supplemental Figure S2

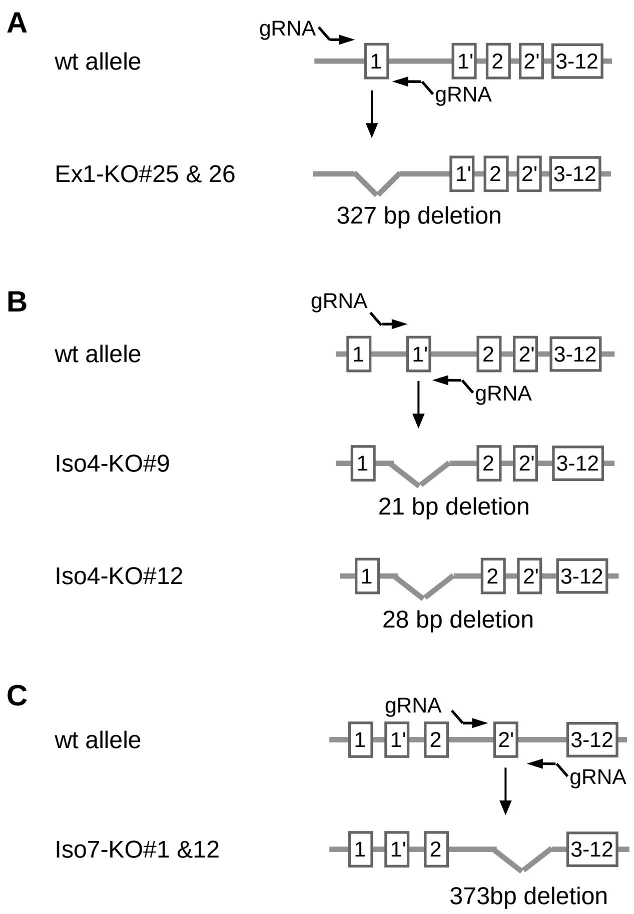

Supplemental Figure S3

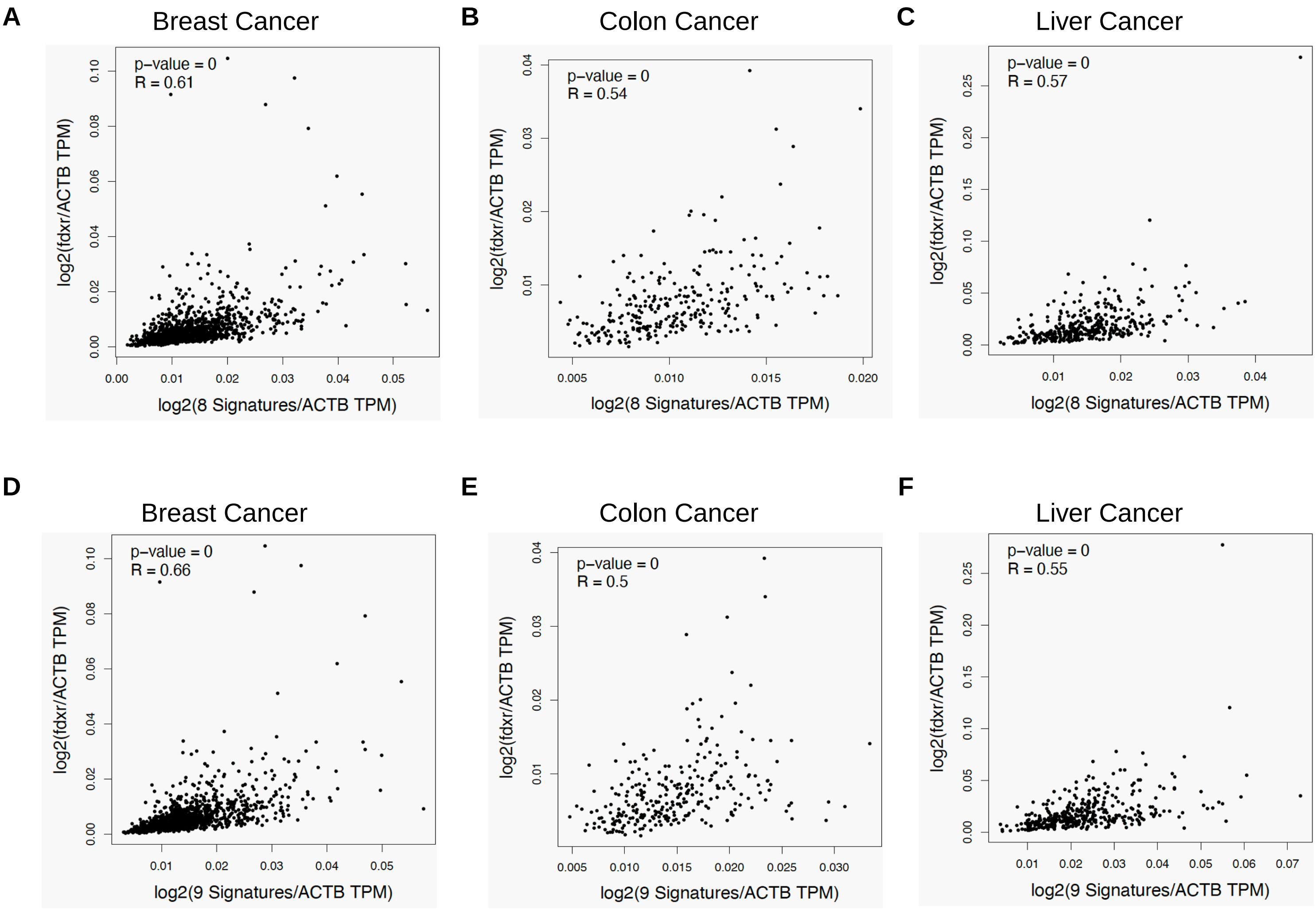

Supplement: Supplementary file 1 [file cells-15-00062-s001.zip › cells-4013477-supplementary.pdf]
